# Report of a series of 82 cases of Buruli ulcer from Nigeria treated in Benin, from 2006 to 2016

**DOI:** 10.1371/journal.pntd.0006358

**Published:** 2018-03-09

**Authors:** Gilbert Adjimon Ayelo, Esai Anagonou, Anita Carolle Wadagni, Yves Thierry Barogui, Ange Dodji Dossou, Jean Gabin Houezo, Julia Aguiar, Roch Christian Johnson, Raoul Saizonou, Kingsley Asiedu, Ghislain Emmanuel Sopoh

**Affiliations:** 1 Centre de Dépistage et de Traitement de l’Ulcère de Buruli d’Allada, Ministry of Health, Allada, Bénin; 2 Programme National de Lutte contre la Lèpre et l’Ulcère de Buruli, Ministry of Health, Cotonou, Bénin; 3 Centre de Dépistage et de Traitement de l’Ulcère de Buruli de Lalo, Ministry of Health, Lalo, Bénin; 4 Centre de Dépistage et de Traitement de l’Ulcère de Buruli de Zagnanado, Ministry of Health, Zagnanado, Bénin; 5 Fondation Raoul Follereau, Paris, France; 6 Centre Inter Facultaire de Formation et de Recherche en Environnement pour le Développement Durable, University of Abomey-Calavi, Abomey-Calavi, Bénin; 7 World Health Organization, Department of Neglected Tropical Diseases, Geneva, Switzerland; 8 Institut Régional de Santé Publique, University of Abomey-Calavi, Ouidah, Bénin; Kwame Nkrumah University of Science and Technology, GHANA

## Abstract

**Background:**

Nigeria is one of the countries endemic for Buruli ulcer (BU) in West Africa but did not have a control programme until recently. As a result, BU patients often access treatment services in neighbouring Benin where dedicated health facilities have been established to provide treatment free of charge for BU patients. This study aimed to describe the epidemiological, clinical, biological and therapeutic characteristics of cases from Nigeria treated in three of the four treatment centers in Benin.

**Methodology/Principal findings:**

A series of 82 BU cases from Nigeria were treated in three centres in Benin during 2006–2016 and are retrospectively described. The majority of these patients came from Ogun and Lagos States which border Benin. Most of the cases were diagnosed with ulcerative lesions (80.5%) and WHO category III lesions (82.9%); 97.5% were healed after a median hospital stay of 46 days (interquartile range [IQR]: 32–176 days).

**Conclusions/Significance:**

This report adds to the epidemiological understanding of BU in Nigeria in the hope that the programme will intensify efforts aimed at early case detection and treatment.

## Author summary

Buruli ulcer (BU) is a neglected tropical disease that mainly affects the skin. The disease results from infection with *Mycobacterium ulcerans*, an environmental bacterium. In Benin, the BU treatment centres usually receive patients from Nigeria. In 2014, a study from one of the treatment centres (CDTUB, Pobe) which borders south-western Nigeria reported on a cohort of 127 PCR-confirmed cases between 2005 and 2013. We describe the epidemiological, clinical, biological and therapeutic characteristics of BU cases from Nigeria treated in the three other CDTUBs.

## Introduction

Buruli ulcer (BU) is a neglected tropical disease that mainly affects the skin. The disease results from infection with *Mycobacterium ulcerans*, an environmental bacterium. BU is found in often swampy and humid areas. The mode of transmission remains obscure to this day, although several hypotheses have been proposed. Many authors have discussed potential reservoirs as well as vectors and transmission mechanisms that vary from region to region depending on the epidemiological, social and local environmental context. Direct human to human transmission of *M*. *ulcerans* is a rare possibility [1]. The main hypothesis is that the surface of the patient’s skin was contaminated by bacteria from an environmental source (e.g. swamps) and introduced into the skin by trauma. It is assumed that insects (aquatic bugs and mosquitoes) are the host and vector of *M*. *ulcerans*. Several experimental and environmental studies have demonstrated the implication of aquatic bugs in transmission of the disease [2–4]. DNA of *M*. *ulcerans* was detected in mosquitoes collected in Australia but a field study conducted in Benin suggested that mosquitoes do not play a central role in the ecology and transmission of *M*. *ulcerans* [5]. Fish has also been identified as a passive reservoir of *M*. *ulcerans* but generally not responsible for direct transmission of the disease [6]. *Acanthamoeba* species have also been identified as natural hosts of *M*. *ulcerans* and have been suggested as responsible for transmission of the disease [7]. No definitive conclusion has yet been drawn about how the disease is transmitted. The World Health Organization (WHO) has classified BU as a neglected tropical disease [8–11]. BU is the third most common mycobacterial infection in the world among immunocompetent individuals after tuberculosis and leprosy [12]. BU is characterized by a chronic necrosis of subcutaneous tissues, ranging from a simple nodule to a large cutaneous ulceration. Sometimes the bone is affected and the resulting damage can impair the functional mobility of the affected limb. Without early and effective treatment, the disease can progress and cause cosmetic complications and sequelae or functional limitations [13] with attendant stigma and social problems [14,15]. It can even lead to limb amputation.

WHO classifies BU lesions into three categories according to severity [16,17]. Category I lesions are single small lesions (e.g. nodules, papules, plaques and ulcers < 5 cm in diameter). Category II lesions consist of non-ulcerative or ulcerative plaques, oedematous forms and single large ulcerative lesions of 5–15 cm in cross-sectional diameter, while lesions in the head and neck regions and the face, disseminated and mixed forms including osteomyelitis, and extensive lesions of more than 15 cm are considered as Category III.

BU mostly affects poor people in rural areas with limited access to health care [11,18–20]. Children aged < 15 years are most commonly affected by the disease [21]. Worldwide, BU has been reported in > 33 countries, mostly within the tropical areas [22]. The majority of BU cases occur in Africa; however, cases have been reported in Australia, French Guiana, Peru and Papua New Guinea [23].

Recognizing that BU constitutes an emerging public health threat, in 1998 WHO established the Global Buruli Ulcer Initiative (GBUI) to coordinate control and research activities worldwide [9,18,22–24].

Up until 2004, the only curative treatment for BU was surgery, which consisted of wide excision to remove all infected tissues including some of the adjacent healthy tissues. Large lesions require skin grafting [18,21,23]. Scientific studies have shown the effectiveness of using different combinations of antibiotics to treat BU [25–27]. The standard treatment since 2004 has been the combination of rifampicin and streptomycin [16]. WHO has issued a provisional recommendation to use the new combination full oral therapy [28]. The introduction of antibiotic therapy has reduced the number of surgical procedures and recurrence rates. Almost all Category I and some Category II lesions can be cured without surgery [29].

In Benin, the BU control programme is known as the *Programme National de Lutte contre la Lèpre et l’UB (PNLLUB)*. It coordinates BU control activities through four care facilities known as *Centres de Dépistage et de Traitement de l’Ulcère de Buruli (CDTUB)*, located in the southern departments where BU is endemic [30].

During the course of their activities, the CDTUBs also receive patients from Nigeria. BU cases were officially reported in Nigeria in 1967 [31] and in 1976 [32] in different Nigerian states. Between 1998 and 2000, BU cases at the Leprosy and Tuberculosis Hospital in Moniaya-Ogoja were confirmed by the Institute of Tropical Medicine, Antwerp, Belgium. In 2006, the Nigerian authorities, in collaboration with a team from Benin and WHO, conducted an assessment of the BU situation in order to identify the endemic areas in Nigeria. The assessment covered only 5 states and therefore could not identify all the endemic regions as originally planned [33]. That was the first time in 2006 when Nigeria notified 9 BU cases to WHO. During 2009–2016, Nigeria reported 511 cases to WHO with increasing numbers of cases each year [34]. BU is known to be endemic in the south of Nigeria mainly in states such as Akwa Ibom, Anambra, Benue, Cross River, Ebonyi, Enugu, Ogun and Oyo [33,35].

Ogun State is divided by two drainage basins, the Yewa and the Ogun rivers, which discharge in separate lagoons. South-west Nigeria is characterized by tropical rainforest, similar to the environments where BU occurs in endemic areas of West Africa [35,36].

In order to establish an effective BU control system in Nigeria, it is imperative that all endemic areas are identified; hence the necessity of providing data on the states where Nigerian BU patients treated in Benin have come from. In 2014, a study from CDTUB in Pobè, Benin reported 127 PCR-confirmed cases of Nigerian BU patients treated in the facility [35]. Pobè is a town on the border with south-western Nigeria, making it the first facility of contact by patients from Nigeria. The objective of our study therefore is to describe the epidemiological, clinical, biological and therapeutic characteristics of BU cases from Nigeria treated in the other three CDTUBs and contribute data to the health authorities in that country.

## Methods

### Study location, type, period and population

This is a retrospective descriptive study of a series of 82 BU cases from Nigeria who were treated in three of the four CDTUBs, namely the CDTUB of Allada, the CDTUB of Lalo and the Centre Nutritionel et Sanitaire Gbemonten (CNSG) of Zagnanado from 1 January 2006 to 31 December 2016.

### Sampling

This was a comprehensive sampling. All the cases from Nigeria, who were clinically suspect for BU and treated in the CDTUBs of Allada, Lalo and Zagnanado during the study period and for whom data were available, were included in this study.

### Variables

For each patient the following information was collected from their medical records and analysed:

- Epidemiological variables: age, gender, state of residence in Nigeria, person who referred them to the CDTUB and delay in seeking treatment.
- Clinical variables: clinical form of the lesion (nodule, plaque, oedema or ulcer), location of the lesion, WHO classification based on category of lesions, restriction or not of joint movement.
- Biological variables were obtained through *IS2404*-PCR results from the Reference Laboratory for Mycobacteria in Cotonou on swab specimens (for ulcerative lesions) or on fine-needle aspiration samples (for non-ulcerative lesions).
- Therapeutic variables: length of hospital stay, treatment outcomes.

### Data sources and statistical methods

The data on the BU cases from Nigeria treated in the three CDTUBs were taken from the PNLLUB’s Register BU 01, which is used to collect standard information for each BU patient. The data were supplemented with additional information from the patients’ medical records kept in each CDTUB. The complete data were recorded with the software Microsoft Excel 2010 and then analysed with the statistical software IBM SPSS Statistics version 20. We only performed a descriptive analysis of the epidemiological, clinical, biological and therapeutic variables of the cases. The maps were drawn from free-access shapefiles obtained from DIVA-GIS (http://www.diva-gis.org/)with QGIS 1.8.0 and ArcView 3.2 software.

### Ethics statement

This retrospective study was conducted after the approval and with the authorization of the Ministry of Health of Benin. The case data in the PNLLUB database used by the authors for this study were anonymized.

## Results

### Socio-demographic, epidemiological characteristics and State of origin of the patients

**Fig 1:**
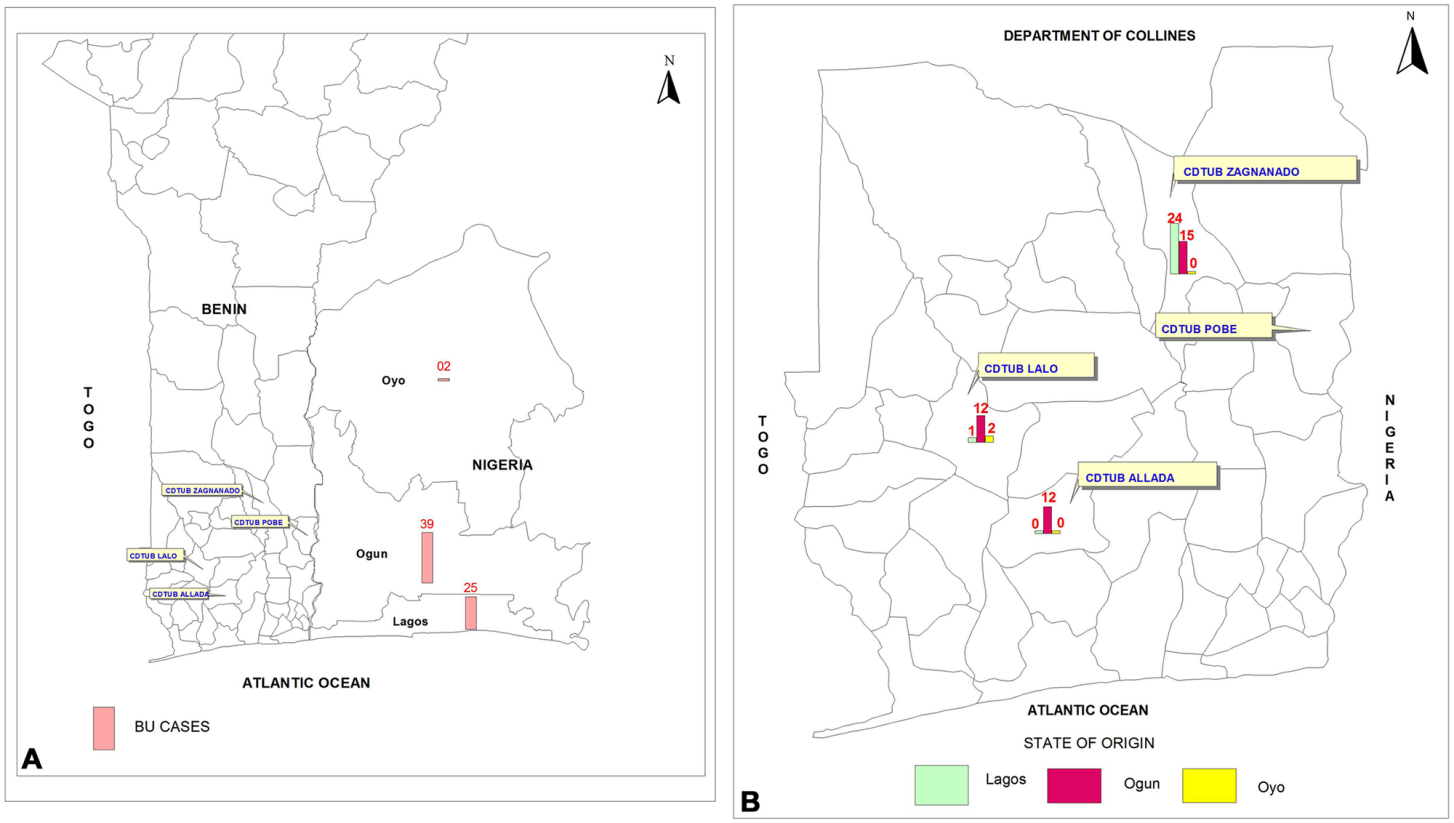
State of origin of the patients and distribution of patients according to the CDTUB in which they were treated. Most of the patients came from Ogun State, which largely borders the south-east of Benin (A). Patients from Ogun State equally seek treatment from all three CDTUBs. Conversely, those from Lagos State mainly go to Zagnanado, the oldest treatment centre in Benin (B).

A total of 82 new patients from Nigeria suspected of having BU were treated in the three Beninese CDTUB from 1 January 2006 to 31 December 2016, with an annual average of 7 patients. Table 1 shows details of the socio-demographic, epidemiological characteristics and states of origin of the patients. Of the 82 patients, 45 (54.9%) were male. The median age of the patients was 20 years (IQR: 13.5–42.5 years). The State of residence in Nigeria was available for 66 patients, of whom 39 (59.1%) were from Ogun State; 25 (37.9%) from Lagos State and 2 (3.0%) from Oyo State. Some 55 patients (67.1%) were treated in the CDTUB of Zagnanado; 15 (18.3%) in the CDTUB of Lalo and 12 (14.6%) in the CDTUB of Allada. Patients from Lagos State were mostly treated in the CDTUB of Zagnanado while those from Ogun State were equally treated in the three centres (Fig 1). The person who referred the patient to the CDTUB was specified for 79 patients; 62 (78.5%) were referred by former BU patients from Nigeria who had been treated in Benin. The other patients were either referred by health agents or by family members. The median delay before seeking medical assistance was 203 days (IQR: 87.5–1638).

**Table 1:** Description of BU cases from Nigeria treated at the CDTUBs of Allada, Lalo and Zagnanado in Benin, N = 82 (2006–2016).

Variables	All CDTUB (N = 82)	CDTUB Allada (N = 12)	CDTUB Lalo (N = 15)	CDTUB Zagnanado (N = 55)
**Median age (years)**	20 (IQR: 13.5–42.5)	10.5 (IQR: 7–19.5)	13 (IQR: 8–17)	27 (IQR: 17–50)
**Gender**				
** Male n (%)**	45 (54.9)	6 (50.0)	10 (66.7)	29 (52.7)
** Female n (%)**	37 (45.1)	6 (50.0)	3 (33.3)	26 (47.3)
**State of residence in Nigeria (16 missing values)**				
Ogun State **n (%)**	39 (59.1)	12 (100.0)	12 (80.0)	15 (38.5)
Lagos State **n (%)**	25 (37.9)	0 (0.0)	1 (6.7)	24 (61.5)
Oyo State **n (%)**	2 (3.0)	0 (0.0)	2 (13.3)	0
**Referral person (3 missing values)**				
Former BU patient **n (%)**	62 (78.5)	7 (70.0)	1 (7.4)	54 (98.2)
Self-referral or family member **n (%)**	11 (13.9)	1 (10.0)	9 (64.0)	1 (1.8)
Health worker **n (%)**	6 (7.6)	2 (20.0)	4 (28.6)	0 (0.0)
**Median Delay in seeking medical assistance in days (2 missing values)**	203 (IQR: 87.5–1638)	91 (IQR: 63–147)	168 (IQR: 84–224)	728 (IQR: 168–2184)
**Clinical Form of lesion**				
Ulcerative form **n (%)**	66 (80.5)	12 (100.0)	14 (93.3)	40 (72.7)
Non-ulcerative form **n (%)**	11 (13.3)	0 (0.0)	0 (0.0)	11 (20.0)
Osteomyelitis **n (%)**	5 (6.1)	0 (0.0)	1 (6.7)	4 (7.3)
**Location of lesion**				
Lower limb **n (%)**	53 (64.6)	2 (16.7)	7 (46.7)	44 (80.0)
Upper limb **n (%)**	22 (26.8)	8 (66.7)	8 (53.3)	6 (10.9)
Multiple locations **n (%)**	4 (4.9)	2 (16.7)	0 (0.0)	2 (3.6)
Other locations **n (%)**	3 (3.7)	0 (0.0)	0 (0.0)	3 (5.5)
**WHO Category of lesion**				
Category I **n (%)**	1 (1.2)	0 (0.0)	0 (0.0)	1 (1.8)
Category II **n (%)**	13 (15.9)	0 (0.0)	5 (33.3)	8 (14.6)
Category III **n (%)**	68 (82.9)	12 (100.0)	10 (66.7)	46 (83.6)
**Restricted joint movement (4 missing values) n (%)**				
** Yes n (%)**	24 (30.8)	11 (91.7)	6 (40.0)	7 (13.7)
** No n (%)**	54 (69.2)	1 (8.3)	9 (60.0)	44 (86.3)
**Number of Patients sampled n (%)**	47 (57.3)	12 (100.0)	12 (80.0)	23 (41.8)
**Patient confirmed by PCR n (%)**	36 (43.9)	12 (100.0)	10 (66.7)	14 (25.5)
**Median Length of hospital stay in days (6 missing values)**	46 (IQR: 32–176)	197 (IQR: 184–318)	256 (IQR: 170–340)	35 (IQR: 27–48)
**Surgical treatment**				
** Yes n (%)**	80 (97.6)	11 (91.7)	14 (93.3)	55 (100.0)
** No n (%)**	2 (2.4)	1 (8.3)	1 (6.7)	0 (0.0)
**Treatment outcomes (2 missing values)**				
Healed **n (%)**	78 (97.5)	11 (91.7)	13 (92.9)	54 (100.0)
Other (Dead, loss to follow up) **n (%)**	2 (2.5)	1 (8.3)	1 (7.1)	0 (0.0)

### Clinical features of the lesion

**Fig 2:**
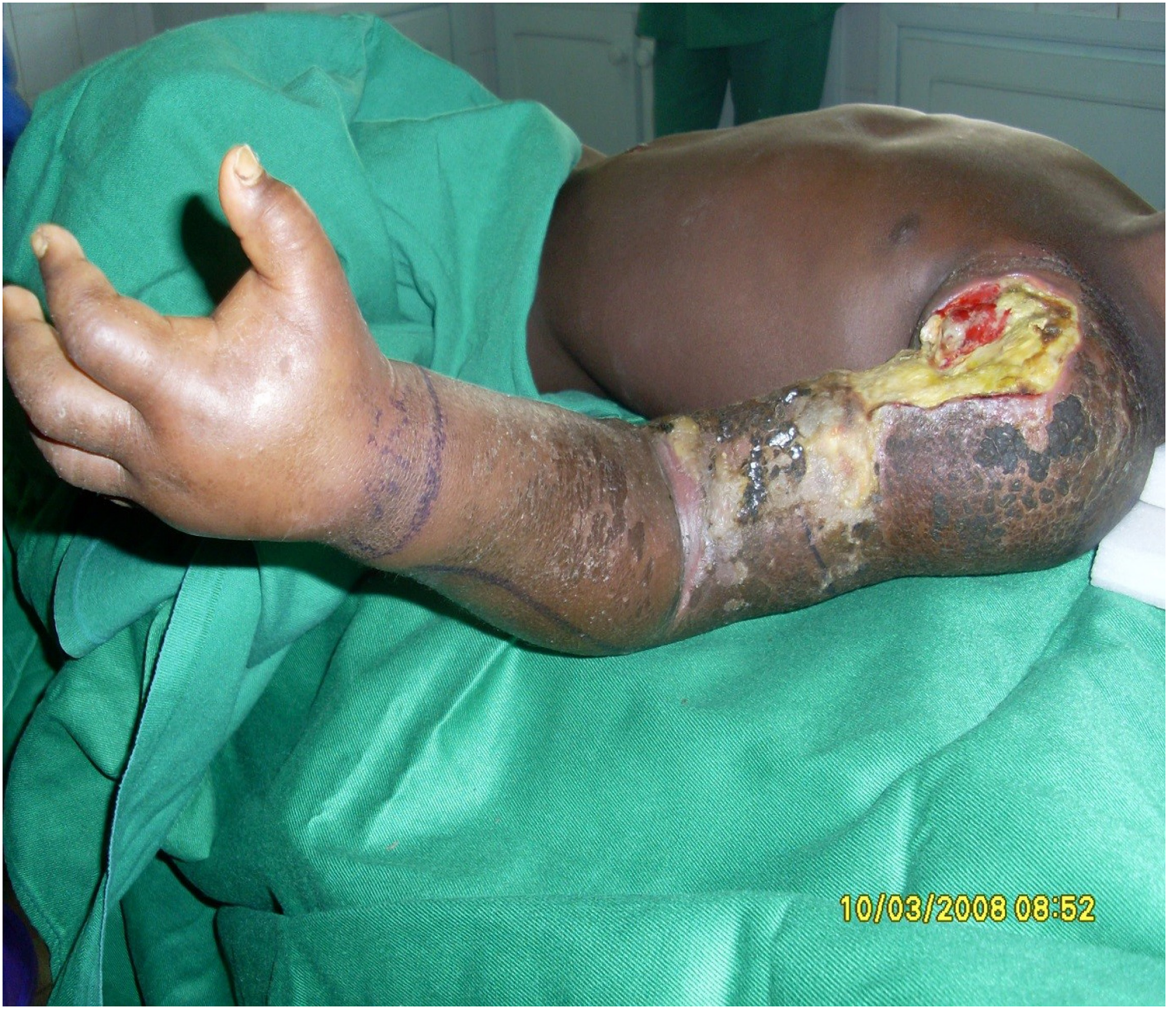
Patient from Nigeria with Category III BU lesions.

Clinically, 66 (80.5%) patients had ulcerative lesions; 11 (13.4%) had nonulcerative lesions (plaque, nodule, oedema) and 5 (6.1%) had osteomyelitis. Of the 82 patients, 53 (64.6%) had lesions on their lower limbs; 22 (26.8%) had lesions on their upper limbs; 3 (3.6%) had lesions on other parts of their body (abdomen, back, head/neck); and 4 (4.9%) had lesions on multiple parts of their body. Some 68 patients (82.9%) had Category III lesions (multiple lesions or lesions > 15 cm in diameter) (Fig 2), 13 (15.9%) had Category II lesions (lesions 5–15 cm in diameter or on their faces/breast/genitalia); only one patient (1.2%) had a Category I lesion (lesion < 5 cm in diameter). For 78 patients, the medical records of 78 patients mentioned whether or not the movements of the affected part were limited at the time of diagnosis; 24 (30.8%) had restricted joint movements.

### Biological, laboratory confirmation

Samples were taken from 12 patients (100.0%) in Allada, 12 patients (80.0%) in Lalo and 23 patients (41.8%) in Zagnanado giving a total of 47 out of the 82 patients (57.3%) for PCR confirmation; 36 out of 47 tested positive.

### Treatment outcome

All the 82 patients received treatment free of charge including specific antibiotic therapy of combined rifampicin with streptomycin for 8 weeks as recommended by WHO as well as surgery and physiotherapy as required. However, 80 patients (97.6%) received surgery. The median length of hospital stay was 46 days (IQR: 32–176 days). The length of hospital stay was relatively longer for the patients treated in the CDTUB of Allada with a median of 197 days (IQR: 184-318) and the CDTUB Lalo with a median of 256 days (IQR: 170–340), while the median length of hospital stay was 35 days (IQR: 27–48) in Zagnanado. Some 78 patients (97.5%) were healed among whom two patients (2.4%) healed without surgery; one patient (1.2%) died during his hospital stay and one patient (1.2%) was lost to follow up. Treatment outcome data were missed for two patients.

The epidemiological, clinical, biological and therapeutic characteristics of the patients are summarized in Table 1.

## Discussion

The objective of our study was to describe the BU cases from Nigeria treated in CDTUBs of Allada, Lalo and Zagnanado, to supplement the previous data published from CDTUB, Pobè in 2015 [35]. Unlike other BU endemic countries where control programmes were started after the International Conference on Buruli ulcer Control and Research (Yamoussoukro, Côte d’Ivoire, 1998) [24], Nigeria’s BU control programme is still in its infancy and does not yet cover all the endemic areas of the country [37]. As a result, BU patients in certain areas of Nigeria use the BU care facilities in neighbouring countries such as Benin and Cameroon [35].

From 2006 to 2016, 82 patients from Nigeria who were clinically suspected for BU were treated free of charge in the CDTUBs of Allada, Lalo and Zagnanado in southern Benin. This number is smaller than the 127 PCR-confirmed BU cases from Nigeria who were treated in the CDTUB Pobè from 2005 to 2013. [35]. Pobè is a town in Benin that borders Nigeria, making it geographically accessible to patients from that country. This explains why the majority of BU cases from Nigeria are treated in Pobè. CDTUB Zagnanado is the oldest BU facility and, after Pobè, the closest CDTUB to Nigeria in Benin. This could explain why most of the cases described in our study were treated in Zagnanado.

Although known as a BU endemic country along with other African countries, it was only in 2009 that Nigeria started regularly reporting BU cases to WHO. From 2009 to 2016, 511 BU cases were reported by Nigeria [34]. However, this figure did not represent the actual incidence of the disease in Nigeria during that period. Together with the publication from Pobè, a total of 209 cases have been documented in Benin.

More than half of our BU patients are aged > 15 years, contrary to the literature which describes children aged < 15 years as being the most affected by the disease and as usually accounting for more than half of the cases reported in Africa [18,19,38]. In this study about Nigeria cases, less than half of the patients (41.7%) were aged < 15 years [39]. This result is similar to that observed by other authors in Nigeria in 2016 [40–42]. It cannot, however, be interpreted as BU mainly affecting older people in Nigeria since the opposite result was observed from the previous study in Pobè among the PCR-confirmed BU cases from Nigeria [35].

The majority of the patients in our study came from Ogun and Lagos, two states in the south-west of Nigeria that border the south-east of Benin. Several authors have also described BU cases in these states, thus confirming that BU may be highly endemic in these regions [33,40–42]. However, Ogun and Lagos states do not have any specialized BU care facilities [33,35,37]. It is therefore essential to develop BU control mechanisms at the local level in these two states to respond to this public health threat. Moreover, an assessment should be conducted in Oyo State in order to understand their epidemiological BU situation.

Contrary to what was observed in the Pobè study [35], there were more male patients in our sample, although gender difference among BU patients has not been described in past literature [18,19]. Here, this difference would be attributable to a random effect or to chance.

The median delay before seeking medical assistance was 203 days or approximately 8 months. This long delay prior to seeking medical attention was also described in the previous study in Pobè in 2015: in their study 24% of patients sought medical help after one year [35]. Another publication by authors from Nigeria in 2016 also described a relatively long delay (median of 16 weeks) prior to seeking medical treatment in a prospective study conducted between May 2014 and September 2015 in 4 BU endemic Nigerian states [39]. Several factors could be the root of cause of the patients’ long delay in seeking medical help. Some studies have shown that geographical inaccessibility to healthcare services is responsible for late recourse to medical care [43,44].

From a clinical perspective, our patients were mostly those with ulcerative lesions and Category III lesions (> 15 cm in diameter). The same results were obtained in the Pobè study [35] as well as by other authors who studied BU cases in Nigeria [38,40]. This could be explained by the patients’ long delay before seeking medical attention, by which time the lesions have had sufficient time to extend. This falls short of the objectives set by WHO, which called upon countries endemic for BU to conduct community actions in order to reduce the proportion of ulcerative cases to < 60% and of Category III cases to < 30% [45]. As the biological confirmation was not systematic at the CDTUB in Zagnanado, the majority of patients were not sampled. Because this facility has lengthy experience in managing the disease by the same team, patients are sampled only in case of doubt about their clinical diagnosis [46], However, PCR confirmation of all suspected BU cases is recommended in order to limit the inappropriate use of specific antibiotic and to ensure standardization of surveillance data across all treatment centres.

The majority of our patients were referred by former BU patients from Nigeria who had been treated in Benin. This confirms the finding that former patients are as effective as community relays, as described previously [47,48], and could therefore be useful in health education and active searches for BU cases in their respective communities.

Unlike the CDTUB of Zagnanado where the median length of hospital stay is 35 days, the lengths of hospital stay were relatively longer in the CDTUBs of Allada with a median of 197 days and Lalo with a median of 256 days. This difference is explained by the fact that, in the CDTUB of Allada and Lalo, patients benefitted from two full months of antibiotic treatment before any surgical procedure. This is the opposite in Zagnanado. The lengthy hospital stay observed in our study, particularly in Allada and Lalo, is linked to the size of the lesions, which are for the most part Category III lesions. Sarfo et al. observed the same connection in 2010 [49]. The late recourse to medical care is another factor that can explain the lengthy hospital stay. In a study in Ghana, a significant association between the length of hospital stay and the size of the lesion was also found [50].

### Conclusion

In addition to local BU cases, the CDTUB of Benin receive BU cases from Nigeria, most of whom are advanced Category III lesions whose care requires more time as well as more material and financial resources and have a socioeconomic impact on both the patients and their caregivers. However, all the treatment was provided for free. These patients mainly come from Nigerian states that border Benin and are referred by former patients who received care from one of the CDTUBs. It is therefore important that BU control activities be intensified in these different states in order to detect cases early and reduce the severity of the disease.

## Supporting information

S1 Dataset Study dataset (table). (XLS)
